# Diagnosis and Management of Diffuse Large B-Cell Lymphoma: Society of Medical Oncology, Pakistan Society of Hematology, and Pakistan Society of Clinical Oncology Joint Clinical Practice Guideline

**DOI:** 10.1200/GO.21.00320

**Published:** 2021-12-13

**Authors:** Raheel Iftikhar, Muhammad Ayaz Mir, Munira Moosajee, Kamran Rashid, Syed Waqas Bokhari, Ahmed Nadeem Abbasi, Tahir Sultan Shamsi, Parvez Ahmed, Hafeez Ud Din, Qamar-un-nisa Chaudhry, Imran N. Ahmad, Mohammad Usman Shaikh, Natasha Ali, Muhammad Umair, Amjad Khan, Mussavir Bangash, Usman Ahmad, Wasim Sattar, Anum Zargham, Azhar Shafi, Ghassan U. Shamshad, Qurratulain Rizvi, Syed Muhammad Irfan, Uzma Zaidi, Naeem Naqi, Humera Mahmood, Asghar Hussain, Ahmed Ijaz Masood, Neelam Siddiqui, Misbah Masood, Mohammad Faheem, Salman Naseem Adil, Zeba Aziz

**Affiliations:** 1 Armed Forces Bone Marrow Transplant Centre, Rawalpindi, Pakistan; 2 Shifa International Hospital, Islamabad, Pakistan; 3 The Aga Khan University Hospital, Karachi, Pakistan; 4 Rashid Nursing Home and Cancer Clinic, Rawalpindi, Pakistan; 5 Shaukat Khanum Memorial Cancer Hospital and Research Centre, Lahore, Pakistan; 6 National Institute of Blood Disease and Bone Marrow Transplantation, Karachi, Pakistan; 7 Quaid e Azam International Hospital, Islamabad, Pakistan; 8 Armed Forces Institute of Pathology, Rawalpindi, Pakistan; 9 Combined Military Hospital, Rawalpindi, Pakistan; 10 Combined Military Hospital, Multan, Pakistan; 11 Ibn e Sina Hospital, Multan, Pakistan; 12 Fauji Foundation Hospital, Rawalpindi, Pakistan; 13 Liaquat National Hospital, Karachi, Pakistan; 14 Hameed Latif Hospital, Lahore, Pakistan; 15 Nuclear Medicine, Oncology and Radiotherapy Institute, Islamabad, Pakistan; 16 Kiran Cancer Hospital, Karachi, Pakistan; 17 Nishtar Medical University, Multan, Pakistan; 18 Inmol Cancer Hospital, Lahore, Pakistan

## Abstract

Diffuse large B-cell lymphoma (DLBCL) is the commonest non-Hodgkin lymphoma encountered by hematopathologists and oncologists. Management guidelines for DLBCL are developed and published by countries with high income and do not cater for practical challenges faced in resource-constrained settings. This report by a multidisciplinary panel of experts from Pakistan is on behalf of three major national cancer societies: Society of Medical Oncology Pakistan, Pakistan Society of Hematology, and Pakistan Society of Clinical Oncology. The aim is to develop a practical and standardized guideline for managing DLBCL in Pakistan, keeping in view local challenges, which are similar across most of the low- and middle-income countries across the globe. Modified Delphi methodology was used to develop consensus guidelines. Guidelines questions were drafted, and meetings were convened by a steering committee to develop initial recommendations on the basis of local challenges and review of the literature. A consensus panel reviewed the initial draft recommendations and rated the guidelines on a five-point Likert scale; recommendations achieving more than 75% consensus were accepted. Resource grouping initially suggested by Breast Health Global Initiative was applied for resource stratification into basic, limited, and enhanced resource settings. The panel generated consensus ratings for 35 questions of interest and concluded that diagnosis and treatment recommendations in resource-constrained settings need to be based on available resources and management expertise.

## INTRODUCTION

Diffuse large B-cell lymphoma (DLBCL) is the commonest non-Hodgkin lymphoma encountered by hematopathologists and oncologists.^1,2^ A study from Southern Pakistan reported higher DLBCL frequency (76.4%) and a younger median age (47.2 years) among newly diagnosed non-Hodgkin lymphoma patients compared with the Western data.^3^ Management guidelines for DLBCL are developed and published by countries with high income and do not cater for practical challenges faced in the low-resource countries. This report by a multidisciplinary panel of experts from Pakistan is on behalf of three major national cancer societies: Society of Medical Oncology Pakistan, Pakistan Society of Hematology, and Pakistan Society of Clinical Oncology. The aim is to develop a practical and standardized guideline for managing DLBCL in Pakistan, keeping in view local challenges which are similar across most of the low- and middle-income countries across the globe.

**CONTEXT**

**Key Objective**
To develop management guidelines for patients with diffuse large B-cell lymphoma (DLBCL), keeping in view the challenges faced in resource-limited countries.
**Knowledge Generated**
This report by a multidisciplinary panel of experts from Pakistan is on behalf of three major national cancer societies, namely Society of Medical Oncology Pakistan, Pakistan Society of Hematology, and Pakistan Society of Clinical Oncology. Consensus guidelines are generated for diagnosis and management of patients with newly diagnosed and relapsed refractory DLBCL patients.
**Relevance**
Management guidelines for DLBCL are developed by countries with high income and do not cater for practical challenges faced in resource-constrained settings. These guidelines are developed keeping in view local challenges, which are similar across most of the low- and middle-income countries across the globe.

### Methodology

The modified Delphi method was used to generate consensus statement as recommended by the ASCO.^4-6^ Literature search was done using PubMed, Embase, and Web of Science. A steering committee comprising seven members reviewed the evidence and drafted initial recommendations after appropriate rationale. A guideline panel comprising 23 experts was formed, which rated the guidelines forwarded by the steering committee on a five-point Likert scale (strongly agree = 1 point to strongly disagree = 5 points). The ratings were accepted if consensus ≥ 75% was achieved.

We used the resource grouping suggested by Breast Health Global Initiative^7^ and applied this to DLBCL. Resource environments were divided into three categories as per resource availability.

1. Basic: Basic-level services are typically provided in a single clinical interaction and include patient review by general practitioners and nononcology fellows.
2. Limited: In addition to basic resources, these are second-tier services that may involve multiple clinical interactions. The aim is timely and accurate diagnosis and to proceed with evidence-based treatments with an aim to produce major improvements in outcome.
3. Enhanced: In addition to basic and limited resource level services, enhanced services will provide third-tier diagnostic and management services required for patients requiring high-intensity chemotherapy and hematopoietic stem-cell transplant (HSCT) for relapsed-refractory or high-risk patients.

Detailed distribution of resource environments as per resource availability is summarized in Table 1. Level of evidence and grading recommendations^8^ are listed in Table 2. Summary of resource-guided interventions is mentioned in Table 3.

**TABLE 1:**
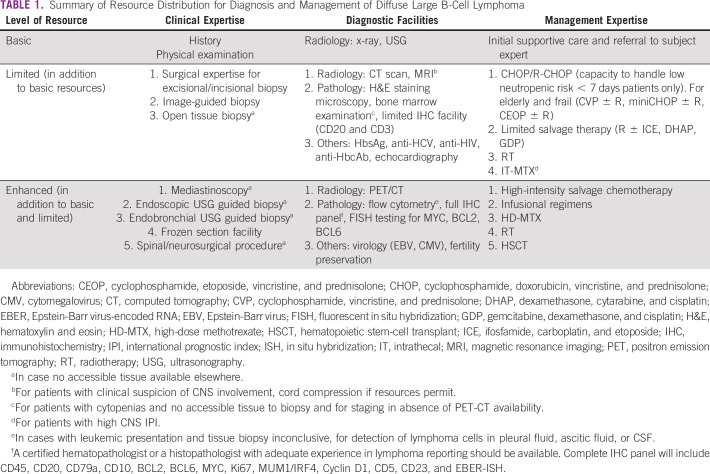
Summary of Resource Distribution for Diagnosis and Management of Diffuse Large B-Cell Lymphoma

**TABLE 2:**
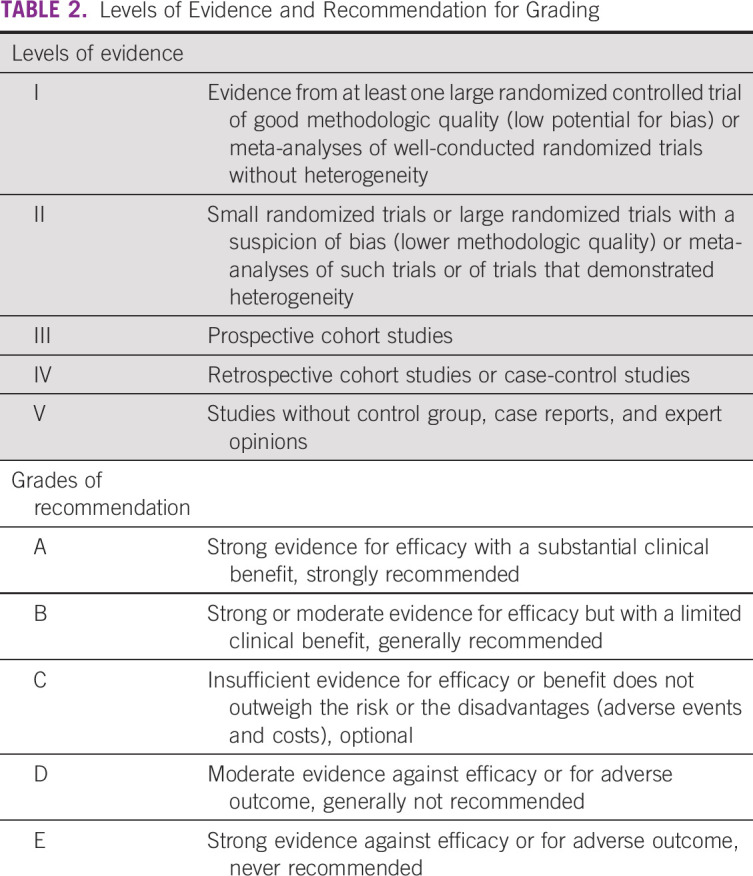
Levels of Evidence and Recommendation for Grading

**TABLE 3:**
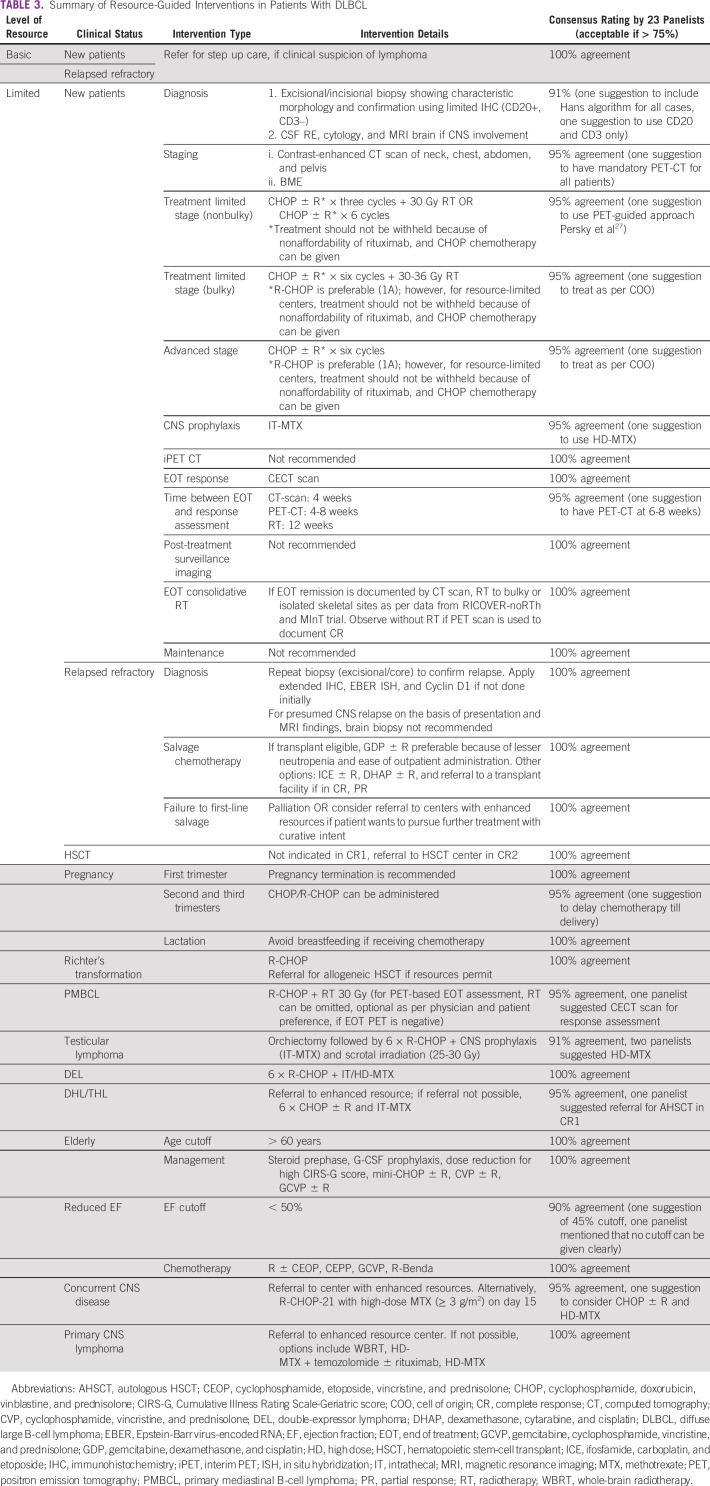
Summary of Resource-Guided Interventions in Patients With DLBCL

## GUIDELINE QUESTIONS

### 1. Diagnosis

#### 1.1. Which essential investigations are required for the diagnosis of DLBCL-not otherwise specified (NOS)?

A surgical excisional biopsy is widely accepted as the gold standard for diagnosis of lymphoma (1A).^9,10^ Core needle biopsies offer an alternative to excisional or incisional biopsy but frequently yield small and insufficient sample. Fine-needle aspiration is discouraged (IIA).^10,11^ For rare cases, bone marrow (BM) examination or microscopic evaluation of pleural fluid, ascitic fluid, and cerebrospinal fluid by cell block histology and confirmation by immunohistochemistry (IHC; limited/extended) or flow cytometry may be required to establish the diagnosis. For cases of suspected primary CNS lymphoma, it is preferable to refer patient to enhanced resource setting for evaluation. Typical morphologic features of DLBCL include complete effacement of normal architecture by sheets of atypical large cells. Tumor cells are large and often resemble centroblasts or immunoblasts. Diagnosis of primary mediastinal B-cell lymphoma (PMBCL) can be made with characteristic clinical presentation and morphology, presence of pan-B markers, and additional staining of CD30, CD200, and TRAF-1.

***Once a diagnosis is given, the patient enters treatment pathway. It is therefore imperative that a correct diagnosis is made within the available resource***.^12^

##### Basic resources

Timely referral to the subject experts for diagnosis and management without advising biopsy or radiologic investigations requiring long waiting times (100% agreement).

##### Limited resources

Excisional/incisional biopsy is preferred. Morphologic diagnosis of DLBCL needs to be confirmed by using limited IHC, CD3, and CD20 (IIIB)^13^; 91% agreement (one suggestion to include Hans algorithm for all cases, one panelist suggested use of only CD3 and CD20 for IHC).

##### Enhanced resources

It is recommended to include CD 3, CD 20, CD 10, BCL6, BCL2, C-MYC, CD 30, Ki-67, MUM1, and cyclin D1^9^ to confirm diagnosis, document cell of origin (COO), and differentiate DLBCL from double-expressor lymphomas (DEL). For characteristic morphology with CD20-negative biopsy, CD138 and leukocyte common antigen are indicated to rule out plasmablastic lymphoma. For cases with immunoblastic morphology, Epstein-Barr virus-encoded RNA in situ hybridization is recommended; 95% agreement (one panelist suggested use of CD3 and CD20 IHC only).

#### 1.2. When and how to differentiate DLBC-NOS from other high-grade lymphomas (including high-grade B-cell lymphoma [HGBCL]-NOS, lymphoblastic lymphoma, Burkitt lymphoma/leukemia [BL], DEL, and double-hit lymphoma).

##### Basic resources

Not applicable.

##### Limited resources

Not recommended routinely. Selected cases at high risk of MYC positivity (ki67% > 90%, early relapse or refractory disease, blastoid morphology, transformation from pre-existing follicular lymphoma) can be referred to enhanced settings; 90% agreement (two suggestions not to do testing for HGBCL and BL).

##### Enhanced resources

If feasible, all DLBCL cases need to be tested for MYC expression and further testing for MYC and BCL2/BCL6 rearrangements in case of MYC expression> 40% and BCL2 > 50% (strong and cytoplasmic). Fluorescent in situ hybridization (FISH) break-apart probes are used, and FISH testing is recommended for all DEL with germinal center-b phenotype and high Ki67 baseline CNS involvement and extensive extra nodal disease (IB)^10^: 90% agreement (two suggestions not to do testing for HGBCL and BL).

#### 1.3. What investigations are required for staging and risk assessment?

#### 1.3.1. What radiologic imaging is required for staging newly diagnosed DLBCL-NOS?

##### Basic resources

Not applicable.

##### Limited resources

Contrast-enhanced computed tomography (CT) scan of the neck, chest, abdomen, and pelvis is recommended. Positron emission tomography (PET) scan can divert resources away from treatment, and careful consideration is required when advising PET-CT scan: 95% agreement (one panelist suggested use of CT scan for all cases).

##### Enhanced resources

PET-CT scan if clinical condition of patient permits (1B)^9,10^: 95% agreement (1 panelist suggested use of CT for all cases).

#### 1.3.2. What is the role of staging BM examination?

##### Basic resources

Not applicable.

##### Limited resources

If CT scan is used for staging, BM examination is needed^10,14^: 95% agreement.

##### Enhanced resources

For patients staged using PET-CT scan, BM testing is not required (2B).^10,14^ For rare patients with suspected low-level BM infiltration (10%-20%), unexplained cytopenias, and discordant lymphoma with negative PET-CT evidence of BM involvement, staging BM examination can be done (2B)^15,16^: 100% agreement.

#### 1.3.3. What additional pretreatment investigations are required for intermediate-high international prognostic index (IPI) patients?

##### Basic resources

Not applicable.

##### Limited resources

Magnetic resonance imaging (MRI) with gadolinium is recommended for patients with neurologic symptoms or signs. Lumbar puncture is recommended for patients with high IPI, advanced-stage disease (Ann Arbor III/IV, BM involvement, > 1 extra nodal site, HGBCL, and those with testicular, breast, and renal involvement [1B]). Intrathecal (IT) chemotherapy needs to be administered at the same time (1A)^10,17^: 95% agreement.

##### Enhanced resources

In addition to evaluation as per limited resources, CSF flow cytometry may be considered (2B)^18,19^: 90% agreement, and two panelists suggested the use of CSF cytology alone.

### 2. Treatment of Newly Diagnosed DLBCL

It is recommended to have hematology oncology multidisciplinary team (MDT) meetings and counseling for fertility preservation in all institutions offering enhanced-level care to patients with lymphoma.^20-22^ If radiotherapy (RT) is part of consensus recommendation of MDT, consultation may be sought from the radiation oncologist before initiation of therapy.^23,24^

#### 2.1. What is the initial treatment of limited-stage (stage IA or IIA) DLBCL?

#### 2.1.1. Limited-stage nonbulky (< 7.5 cm).

##### Basic resource

Not applicable.

##### Limited resources

Cyclophosphamide, doxorubicin, vincristine, and prednisolone (CHOP) ± rituximab (R)* × three cycles + 30 Gy RT OR CHOP ± R* × six cycles.

*R-CHOP is preferable (1A); however, it is not recommended to withhold treatment because of nonaffordability of rituximab, and CHOP chemotherapy can be given^25^: 95% agreement.

##### Enhanced resources

Options include the following:

- Disease at sites with low RT morbidity (groin, neck, and axilla): three cycles of R-CHOP + RT 30 Gy (1B).^9,10^
- For IPI = 0, provide four cycles of R-CHOP followed by two cycles of rituximab if interim PET (iPET) scan document complete metabolic response (Deauville score 1-3).^26^ If IPI > 0 and disease at sites where RT needs to be avoided, provide six cycles of R-CHOP(1A).^10^
- PET-directed therapy using three cycles of R-CHOP followed by iPET assessment. If iPET-negative (Deauville score 0-3), one additional R-CHOP without RT and if iPET-positive (Deauville score 4), an additional cycle of R-CHOP followed by RT 30 Gy and end-of-treatment (EOT) PET scan. For the Deauville score of 5, repeat biopsy is advised^27^ (95% agreement, and one panelist suggested PET-directed therapy for all patients).

#### 2.1.2. Limited-stage bulky (≥ 7.5 cm).

##### Basic resource

Not applicable.

##### Limited resources

CHOP ± R* for six cycles

*R-CHOP is preferable (1A); however, it is not recommended to withhold treatment because of nonaffordability of rituximab, and CHOP chemotherapy can be given (100% agreement).

##### Enhanced resources

R-CHOP for six cycles^9,10,28^: 95% agreement, and one panelist suggested PET-directed therapy for all patients. RT may be considered as per physician and patient discretion keeping in mind the risks and benefits (IIB).

#### 2.2. What initial treatment is recommended for advanced-stage DLBCL?

Over the past decade, the attempts to improve on R-CHOP with dose intensification and incorporation of novel agents have been largely futile.^29-33^

##### Basic resources

Not applicable.

##### Limited resources

CHOP ± R* for six cycles (95% agreement, and one suggestion to treat as per COO)

*R-CHOP is preferable (1A); however, it is not recommended to withhold treatment because of nonaffordability of rituximab, and CHOP chemotherapy can be given.

##### Enhanced resources

R-CHOP for six cycles (1A)^9,10,34^ (95% agreement, and one suggestion to treat as per COO). RT may be considered for initial bulky disease as per physician and patient discretion (IIB).

#### 2.3. What is the optimal CNS prophylaxis for newly diagnosed DLBCL?

##### Basic resources

Not applicable.

##### Limited resources

IT chemotherapy: 100% agreement.^35,36^

##### Enhanced resources

IT chemotherapy or high-dose methotrexate (HD-MTX) as per physician and patient discretion (95% agreement, and one panelist suggested use of IT chemotherapy only).^37,38^ Recent studies by Puckrin et al^35^ and Eyre et al^36^ have called efficacy of HD-MTX for CNS prophylaxis into question by documenting lack of benefit in CNS relapse, overall survival (OS), and disease-free survival with HD-MTX.

#### 2.4. How to monitor patient after EOT?

#### 2.4.1. What radiologic investigation to choose for EOT response?

If resources permit, PET-CT scan at EOT is preferred for response assessment. Pretreatment PET-CT is not essential for documenting EOT response.^12^

##### Basic resources

Not applicable.

##### Limited resources

CT scan for response assessment as per Lugano response assessment criteria^16^: 95% agreement, and one panelist suggested use of PET-CT.

##### Enhanced resources

PET-CT scan: 95% agreement, and one panelist suggested the use of CT.

#### 2.4.2. What is the optimal time between EOT and response assessment?

For patients receiving chemotherapy, CT scan needs to be done after 4 weeks and PET scan 4-8 weeks after treatment: 100% agreement. PET scan is recommended 12 weeks after RT (III B)^39^; 100% agreement.

#### 2.4.3. What post-treatment surveillance is recommended for patients achieving complete remission at EOT (limited-stage and advanced-stage disease)?

For patients achieving complete remission after EOT, guideline recommendations of National Comprehensive Cancer Network (NCCN), British Committee for Standardization in Hematology, and European Society of Medical Oncology guidelines do not recommend routine surveillance scans for stage I/II disease. NCCN recommends only CT imaging every 6 months for 2 years for stage III/IV disease. CT scans are preferred over PET-CT.

##### Basic resources

Not applicable.

##### Limited resources

Surveillance radiologic imaging not recommended if EOT complete remission is documented (IB): 100% agreement.

##### Enhanced resources

No radiologic imaging for limited-stage DLBCL. CT scan every 6 months for 2 years for advanced stage may be considered, at physician discretion (95% agreement, and one panelist suggested no imaging for advanced stage disease).

#### 2.5. What is the role of EOT consolidative RT in patients with bulky disease?

The role of RT following CR (on the basis of CT scan) was documented by German RICOVER-noRTh trial,^28^ where addition of RT improved event-free survival (HR 2.1, *P* = .005) and a trend toward improved OS (HR 1.6, *P* = .127). However, a recent study by Freeman et al^40^ documented that for patients with EOT-negative PET scan, RT can be avoided without increasing relapse risk and compromising OS in patients with bulky diseases at diagnosis.

##### Basic resources

Not applicable.

##### Limited resources

If EOT remission is documented by CT scan, RT to bulky or isolated skeletal sites as per data from RICOVER-noRTh and MInT trial. Observe without RT if PET scan is used to document CR^40^: 100% agreement.

##### Enhanced resources

If EOT remission is documented by CT scan, RT to bulky or isolated skeletal sites as per data from RICOVER-noRTh and MInT trial. Observe without RT if PET scan is used to document CR^40^: 100% agreement.

#### 2.6. What is the role of iPET-CT assessment in limited-stage and advanced-stage DLBCL?

##### Basic resources

Not applicable.

##### Limited resources

Not recommended; 100% agreement.

##### Enhanced resources

Not recommended: 95% agreement, and one suggestion to use iPET scan.

#### 2.7. What is the role of maintenance therapy in DLBCL? When will you offer it?

Elderly patients with DLBCL relapsing after first-line chemoimmunotherapy have limited therapeutic options and poor OS. A phase III randomized REMARC trial documented that lenalidomide maintenance improved PFS versus placebo in elderly patients with DLBCL responding to first-line R-CHOP.^41,42^

##### Basic resources

Not applicable.

##### Limited resources

Not recommended: 100% agreement.

##### Enhanced resources

Lenalidomide maintenance (2B) for patients age 60-80 years^41^ as per physician and patient discretion (86% agreement, and one suggestion to avoid, one suggestion to use in partial response [PR], and one suggestion to use in clinical trial).

### 3. How to Manage Relapsed-Refractory Patients

#### 3.1. What investigations are to be advised for relapsed-refractory patients?

The postpredictive value of a PET-positive lesion is low (50%-82%), and a rebiopsy is strongly recommended before second-line treatment. For patients with presumed CNS relapse on the basis of presentation and supportive MRI findings, repeat brain biopsy is not recommended as it provides little clinical benefit and adds to morbidity and mortality.^43^

##### Basic resources

Not applicable.

##### Limited resources

Repeat biopsy (excisional, core biopsy; 1A), CT scan for staging (95% agreement).

##### Enhanced resources

Repeat biopsy (excisional, core biopsy; 1A), PET-CT (95% agreement, and one suggestion to use PET-CT only if transplant eligible). Extended IHC, cyclin D1, and Epstein-Barr virus-encoded RNA in situ hybridization need to be done.

#### 3.2. What salvage treatment is to be offered for relapsed-refractory patients?

##### Basic resources

Not applicable.

##### Limited resources

If transplant eligible, gemcitabine, dexamethasone, and cisplatin (GDP) ± R preferable because of lesser neutropenia and ease of outpatient administration. Other options include ifosfamide, carboplatin, and etoposide (ICE) ± R, dexamethasone, cytarabine, and cisplatin (DHAP) ± R, and referral to a transplant facility if in CR or PR (95% agreement, and one suggestion to use ICE ± R preferably and use salvage only if transplant eligible).

##### Enhanced resources

ICE ± R, DHAP ± R, and GDP ± R salvage followed by autologous HSCT (AHSCT) consolidation if in CR or PR. Rituximab to be included if disease relapsed after 6 months and rebiopsy shows CD20 expression. For primary refractory DLBCL, rituximab can be omitted in salvage (NCCN ver 4.2021) (95% agreement, and one suggestion to use ICE ± R preferably and use salvage only if transplant eligible).

#### 3.3. What further treatment will you offer to patients not responding to first-line salvage?

##### Basic resources

Not applicable.

##### Limited resources

Option of palliative versus novel agents needs to be discussed depending on transplant eligibility and patient resources/wishes. Referral to centers with enhanced resources if patient wants to pursue further treatment with curative intent: 95% agreement, and one panelist suggested palliation for all cases.

##### Enhanced resources

For patients with available resources and good functional status, options include polatuzumab + bendamustine + rituximab. Patients with MYD-88 mutation have shown impressive responses to ibrutinib. Alternatively , brentuximab vedotin (CD30-positive lymphoma), blinatumomab and tafasiamab can be considered^34^: 91% agreement, and two panelists suggested palliation for all cases.

### 4. What Are Transplant Indications in Patients With DLBCL?

#### 4.1. Transplant indications in newly diagnosed DLBCL.

##### Basic resources

Not applicable.

##### Limited resources

Not recommended in CR1: 100% agreement.

##### Enhanced resources

Not recommended in CR1: 100% agreement.

#### 4.2. Transplant indications in relapsed-refractory DLBCL.

##### Basic resources

Not applicable.

##### Limited resources

Second complete remission (CR2) and beyond: 95% agreement, and one suggestion to offer palliation.

##### Enhanced resources

CR2 and beyond. Some cases can be offered transplant in PR if chemosensitive: 95% agreement, and one suggestion to offer palliation.

#### 4.3. Transplant indications in DHL, DEL, and HGBCL-NOS.

##### Basic resources

Not applicable.

##### Limited resources

(100% agreement)

**DHL/DEL:** Transplant in CR1 is not recommended^44^ (IIB).

##### Enhanced resources

(100% agreement)

**DHL/DEL:** Transplant in CR1 is not recommended^44^ (IIB).

#### 4.4. Till what age will you offer AHSCT to patients with DLBCL with otherwise good functional status and low hematopoietic stem-cell transplant comorbidity index score?

For patients age < 65 years with good performance status, Eastern Cooperative Oncology Group (ECOG) 0-1, and no major organ dysfunction, AHSCT can be considered: 100% agreement.

#### 4.5. What are the indications for allogenic HSCT in DLBCL?

Recommended in the following subsets of patients^45^ (100% agreement):

- Relapsing after autologous HSCT (IIB)
- Those failing to harvest stem cells for autologous transplant (IIB)
- Richter transformation (IIB)

### 5. Management of Special Circumstances

#### 5.1. How will you manage limited-stage and advanced-stage DLBCL in pregnancy?

Management of lymphoma in pregnancy requires management by MDT. Diagnosis and staging of lymphoma require investigations with an aim to minimize radiation exposure to mother. Whole-body MRI, x-ray chest, and ultrasonography are preferable in this context as per resource availability.

##### First trimester

Pregnancy termination is recommended. For selected cases with limited-stage disease and unwilling for termination, watchful waiting till second trimester^46^ (100% agreement).

##### Second and third trimesters

*CHOP/*R-CHOP can be administered after first trimester. Risk of fetal malformations is not increased; however, risk of preterm birth is higher.^47^ Methotrexate needs to be avoided throughout pregnancy: 95% agreement, and one suggestion to delay chemotherapy till delivery.

##### Delivery and lactation

Delivery to be scheduled 2-3 weeks after cycle completion and breastfeeding is discouraged for patients receiving chemotherapy in the last trimester (100% agreement).

#### 5.2. What treatment will you offer to patients with Richter's transformation?

##### Basic resources

Not applicable.

##### Limited resources

R-CHOP 21 and if in CR and transplant eligible, referral for reduced-intensity conditioning allogeneic HSCT consolidation: 95% agreement, and one panelist suggested observation after chemotherapy.

##### Enhanced resources

R-CHOP 21 and if CR after chemoimmunotherapy, consolidation with reduced-intensity conditioning allogeneic HSCT.^48^ For patients not responding to first-line R-CHOP, other options include R-GDP, R-DHAP, and R-ICE. Off-label use of rituximab + lenalidomide + ibrutinib (RLI), checkpoint inhibitors, and venetoclax: 95% agreement, and one panelist suggested observation after chemotherapy.

#### 5.3. What treatment will you offer as upfront therapy for PMBCL.

Because of lack of randomized trials, optimal first-line treatment in patients with PMBCL is unknown. Aviles et al reported the use of RT as adjuvant treatment in patients with CR after six cycles of R-CHOP and documented improvement in the PFS and OS with minimal toxicities.^49^

PET-directed approach to omit RT for EOT PET-negative patients is supported by recent studies^50,51^ and currently being evaluated in the IELSG37 trial. A phase II trial conducted by the National Cancer Institute using DA-EPOCH-R (dose-adjusted etoposide, prednisone, vincristine, cyclophosphamide, doxorubicin, and rituximab) without radiation documented a 5-year event-free survival of 93% among 51 patients.

##### Limited resources

R-CHOP + RT if CT scan is used for remission assessment. For PET-based EOT assessment, RT can be omitted (optional as per physician and patient preference) if EOT PET is negative (41): 95% agreement, and one panelist suggested CT imaging.

##### Enhanced resources

DA-EPOCH-R preferred. The other option is R-CHOP ± RT as per EOT PET response: 95% agreement, and one panelist suggested R-CHOP and CT imaging.

#### 5.4. What treatment will you offer to patients with testicular lymphoma?

Testicular involvement in DLBCL is associated with increased CNS involvement at diagnosis, increased risk of CNS relapse, and adverse prognosis.

##### Limited resources

Orchiectomy followed by six × R-CHOP + CNS prophylaxis (IT-chemotherapy) and scrotal irradiation (25-30 Gy)^52^: 91% agreement, and two panelists suggested HD-MTX.

##### Enhanced resources

Orchiectomy followed by six × R-CHOP + CNS prophylaxis (IT/HD-MTX) and scrotal irradiation (25-30 Gy)^52^: 95% agreement, and one panelist suggest IT chemotherapy.

#### 5.5. What is the optimal first-line treatment for patients with DEL and DHL?

##### Basic resources

Not applicable.

##### Limited resources

**DEL**: six × R-CHOP + ITchemotherapy

**DHL**: Referral to enhanced resource setup for intensive regimens. If referral not possible, it is suggested to give six × R-CHOP with IT chemotherapy (95% agreement).

##### Enhanced resources

**DEL**: six × R-CHOP + IT chemotherapy/HD-MTX

**DHL**: six × DA-EPOCH-R + IT chemotherapy/HD-MTX

(95% agreement, and one panelist suggested R-CHOP and IT chemotherapy for DHL)

#### 5.6. How to manage elderly patients with DLBCL?

#### 5.6.1. What age limit will you consider for classifying patients as elderly in Pakistani population?

Generally, geriatric assessment needs to be done routinely for patients age > 60 years; 100% agreement.

#### 5.6.2. What factors will you consider in deciding upfront treatment (curative *v* palliative)?

Use of ECOG alone is not recommended to decide upfront treatment. Elderly patients with low Charlson Comorbidity Index (CCI) score and low Cumulative Illness Rating Scale-Geriatric (CIRS-G) score are candidates for curative therapy^36^: 95% agreement, and one panelist suggested low-intensity treatment for all patients age > 60 years.

#### 5.6.3. How will you approach management of newly diagnosed elderly patients with DLBCL?

- Consider steroid prephase if ECOG > 2 (2B)
- Primary granulocyte colony-stimulating factor prophylaxis for patients age > 65 years or those who are frail and with significant comorbidities (1A)
- Dose reduction if high CCI or CIRS-G score
- Standard R-CHOP for low CCI and CIRS-G patients age < 70 years
- R-mini-CHOP for patients age > 70 years
- Alternatives include RLI;^53^ bendamustine-rituximab;^54^ R-cyclophosphamide, vincristine, and prednisolone (CVP); R-gemcitabine, cyclophosphamide, vincristine, and prednisolone (GCVP)

(95% agreement, and one suggestion to avoid doxorubicin for patients age > 60 years and use CVP ± R)

#### 5.7. What treatment will you offer to patients with reduced ejection fraction (EF)? What cutoff of EF will you consider for treatment modification?

The definition of chemotherapy-related heart dysfunction is decline in EF of at least 5% to below 55% with accompanying signs or symptoms of congestive heart failure, or a decline in EF of at least 10% to below 55% without accompanying signs or symptoms.^55^ For patients with cardiac dysfunction, substitution of doxorubicin with etoposide, gemcitabine, or liposomal doxorubicin may be considered (IIIC).^56^

#### Basic resources.

Not applicable.

#### Limited resources.

R-cyclophosphamide, etoposide, vincristine, and prednisolone (CEOP); R-cyclophosphamide, etoposide, procarbazine, and prednisolone (RCEPP); and RGCVP. Treatment modification if EF < 50%; 100% agreement.

#### Enhanced resources.

R-CEOP, RCEPP, and RGCVP: treatment modification if EF < 50% (100% agreement).

#### 5.8. How will you manage patients with concurrent CNS disease at presentation?

##### Basic resources

Not applicable.

##### Limited resources

Referral to center with enhanced resources. Alternatively, CHOP-21 ± R with IT chemotherapy and whole-brain radiotherapy (WBRT): 95% agreement, and one suggestion to consider CHOP ± R and HD-MTX.

##### Enhanced resources

Intensive chemoimmunotherapy (methotrexate, thiotepa, rituximab/R-ICE/HD-MTX + ifosfamide alternating with Ara-C+ thiotepa) followed by autologous HSCT/etoposide and cytarabine consolidation (100% agreement).

#### 5.9. What treatment plan would you offer for primary CNS large B-cell lymphoma in first line and relapse setting in young/fit and older/less fit individuals?

##### Newly diagnosed

##### Limited resources

WBRT, HD-MTX + temozolomide ± rituximab, and HD-MTX + procarbazine + vincristine ± rituximab (100% agreement).

##### Enhanced resources

Reasonable regimens include (1) rituximab + HD-MTX + high-dose cytarabine; (2) rituximab + HD-MTX + temozolomide; (3) HD-MTX + Ara-C + thiotepa + rituximab; and (4) rituximab + HD-MTX + procarbazine + vincristine.^57^ There is growing evidence to suggest that the use of AHSCT consolidation improves survival outcomes. Otherwise, consolidation with high-dose cytarabine OR WBRT needs to be considered if AHSCT is not available in patients age < 60 years (100% agreement).

##### Relapsed refractory

Treatment options for relapsed refractory PCNS DLBCL are limited, and outcomes are dismal.

##### Basic resources

Not applicable.

##### Limited resources

WBRT, HD-MTX, high-dose Ara-C and etoposide, lenalidomide, and temozolomide (combination): 91% agreement, and two panelists suggested palliation.

##### Enhanced resources

Reasonable regimens include (1) rituximab + HD-MTX + high-dose cytarabine; (2) high-dose cytarabine with etoposide followed by thiotepa-based AHSCT; (3) ibrutinib; (4) lenalidomide; and (5) immune checkpoint inhibitors followed by AHSCT if in CR: 91% agreement, and two panelists suggested palliation.
